# Exogenous melatonin advances the ram breeding season and increases testicular function

**DOI:** 10.1038/s41598-020-66594-6

**Published:** 2020-06-16

**Authors:** K. R. Pool, J. P. Rickard, T. Pini, S. P. de Graaf

**Affiliations:** The University of Sydney, Faculty of Science, School of Life and Environmental Sciences, Sydney, NSW 2006 Australia

**Keywords:** Animal physiology, Reproductive biology

## Abstract

Governed by melatonin, ovine reproductive seasonality limits production outcomes due to periods of decreased reproductive efficiency. Though it is established that slow-release melatonin implants improve out of season reproductive performance in the ewe, the comprehensive effects of exogenous melatonin in the ram remain inconclusive. This study aimed to ultimately clarify the ability of exogenous melatonin to alter ram reproductive function during the non-breeding season and the subsequent breeding season. Hence, we investigated the effect of exogenous melatonin on reproductive endocrinology, semen quality and production, testicular size and libido in Merino and Poll Dorset rams (n = 31, using a subset of 18 rams for analysis of semen production and quality). Melatonin treatment resulted in elevation of melatonin in seminal plasma from 1–8 weeks post-implantation and in blood plasma at 6 weeks post-implantation. The blood plasma testosterone of implanted rams was greater than controls at both 6 weeks post-implantation and during the following breeding season. Implanted rams exhibited increased testicular size and number of sperm per ejaculate from 3–12 weeks post-implantation but did not demonstrate any change in sperm motility or morphology in response to treatment. Compared to their control counterparts, melatonin-treated Poll Dorset rams exhibited a lower percentage of sperm DNA fragmentation during several weeks of the non-breeding season. Though melatonin increased the likelihood of ejaculate collection in Poll Dorset rams (P < 0.05), libido was otherwise unaffected by treatment. Melatonin did not alter seminal plasma concentrations of inhibin A or Anti-Mullerian hormone, however, for the first time in the ram we have shown Anti-Mullerian hormone to be positively correlated with the number of sperm per ejaculate and sperm motility (*r* = 0.464 and 0.3242 respectively, P < 0.001), and inhibin A to be correlated to the number of sperm per ejaculate (*r* = 0.1786, P = 0.0135). These results indicate that melatonin is able to both systemically upregulate reproduction and act directly upon testicular function in the ram.

## Introduction

As seasonal breeders, the initiation of ovine reproduction is regulated by decreasing photoperiod, translated into a physiological signal by pineal neurohormone melatonin^1,2^ Evidence suggests that melatonin acts upon both the ovine pituitary and hypothalamus, allowing modulation of the hypothalamic-pituitary-gonadal axis for seasonal reproduction^3–5^. Though the degree of reproductive regression varies amongst breeds, anoestrus tends to be more pronounced in the ewe, delineated by a halt in ovarian cycling^6,7^. Whilst the ram retains some fertility in the non-breeding season, this period is characterized by a reduction in libido, testicular size, sperm quality and quantity, subsequently leading to a period of reduced productivity^8,9^. Reproductive seasonality in the ram remains a hindrance in sheep production systems, as it limits reproductive potential, and thus the lifetime production of the animal.

Whether melatonin is able to influence ram fertility through additional mechanisms to photoperiodic translation remains to be elucidated. Melatonin receptors in the ram reproductive tract^10,11^ and on spermatozoa^11–13^ indicate that melatonin likely has other direct roles in the onset and maintenance of seasonal fertility. *In vitro*, the binding of melatonin to receptors on Sertoli cells upregulates Sertoli-cell mediated Leydig cell testosterone production^10^, and binding to melatonin receptors on spermatozoa appears to influence sperm capacitation and acrosome reactivity^14^. Furthermore, increased seminal plasma concentrations of melatonin have been correlated to antioxidant enzyme glutathione reductase (GRD) activity^15^. Melatonin itself is a potent antioxidant, able to scavenge a wide range of reactive oxygen species (ROS) that contribute to oxidative damage in sperm^16^,which may partially explain its beneficial effects upon sperm quality *in vitro* across a range of species^17–20^.

Melatonin is currently utilised commercially to modulate ovine seasonality; in the form of a slow release implant, melatonin is demonstrated to advance the breeding season of the ewe^21–27^ Whilst exogenous melatonin is shown to increase ram testosterone secretion^28–30^ and testicular size^21,28,30,31^, there are conflicting reports upon sperm production and quality^28,29,32,33^ and discrepancies regarding when reproductive changes occur relative to melatonin implantation. Furthermore, it is unclear whether exogenous melatonin exerts a uniform effect across sheep breeds of varying seasonal reproductive regression, which may partially account for inconsistent results between studies.

In previous reports, it difficult to distinguish whether exogenous melatonin merely mimics the effect of decreasing photoperiod, consequently advancing the breeding season, or if there are further beneficial effects upon sperm production and function. Though melatonin-induced changes to sperm production and quality remain debated, hormone production is shown to distinctly vary with melatonin secretion in the ram^15,30,34,35^. Endocrine markers such as testosterone, Anti-Mullerian Hormone (AMH) and inhibin are increasingly explored as indicators of testicular function. In other species, seminal plasma inhibin and AMH concentrations positively correlate with elevated spermatogenesis^36,37^ reduced oxidative stress^36,38^, and improved semen quality^39–43^. In the ram, changes in these endocrine profiles may be similarly correlated with sperm production and quality and could support that exogenous melatonin improves testicular function in the non-breeding season.

In Australia, despite the availability of melatonin implants to advance the breeding season of the ewe, there is no equivalent method to promote ram fertility in the non-breeding season. As past studies do not agree upon the effects of exogenous melatonin in the ram, both natural and artificial breeding remains restricted by the natural reduction in ram libido, semen production and quality in the non-breeding season. In the present study, we aimed to clarify the effects of exogenous melatonin upon ram reproductive parameters throughout the duration of the non-breeding season using two breeds of differing seasonal reproductive regression. To identify any long-term effects of melatonin upon ram reproduction, this study was continued into the breeding season subsequent to melatonin implantation.

## Results

### Melatonin alters ram behaviour and increases scrotal circumference

In Poll Dorset rams, treatment with melatonin increased the likelihood of the ram producing an ejaculate during study weeks 9–12 (P = 0.007, see Supplementary Fig. S1), though did not produce any effect in Merino rams.

There was no effect of treatment in either breed observed upon either weekly or advanced libido score (P > 0.05, data not shown). Regardless of treatment, differences between the non-breeding and breeding seasons were observed in all rams during the advanced libido testing, with increases during the breeding season in the average number of ejaculations (0.73 ± 0.1 vs 2.266 ± 0.2, P < 0.001), times the ram courted the ewe (18.466 ± 2.3 vs 33.14 ± 2.4 P < 0.001) and number of attempted mounts (5.35 ± 1.1 vs 9.375 ± 1.3, P < 0.001) during the 20 minute testing period.

**Figure 1:**
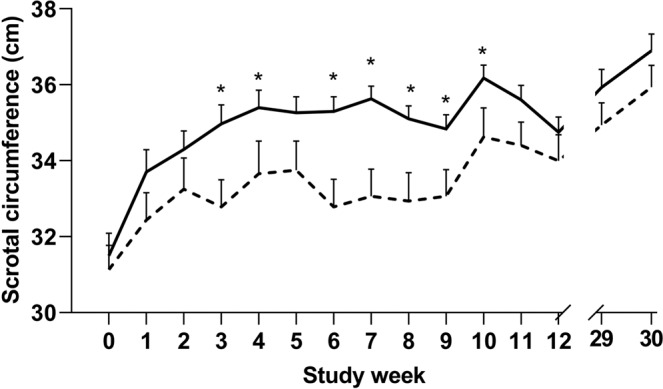
Scrotal circumference of melatonin implanted (-) and control (**-**) Merino and Poll Dorset rams (n = 31) from study week 0 (prior to implantation) to study week 30 (following breeding season). Weekly values are presented as means ± S.E.M. *Indicates significant difference between treatment groups (P < 0.05).

Compared to their control counterparts, melatonin treated rams of both breeds recorded larger scrotal circumferences during study weeks 3–4, 6–10 (P < 0.001, Fig. 1). In melatonin treated rams, scrotal circumference was greater than pre-implantation measurements from study week 3 onwards (P < 0.001) whilst the scrotal circumference of control rams did not increase until the breeding season.

Ram weight varied (P < 0.001) between control and melatonin treatment groups respectively only during study week 11 (90.00 ± 2.2 vs 88.07 ± 1.8 kg), 12 (92.25 ± 2.1 vs 90.07 ± 1.7 kg), 29 (91.00 ± 2.2 vs 95.50 ± 2.3 kg) and 30 (87.97 ± 2.1 vs 90.40 ± 2.1 kg).

There was no effect of treatment upon body condition score (P > 0.05, see Supplementary Fig. S2).

### Ram sperm production, but not quality, is improved following melatonin implantation

In both breeds, the sperm concentration of ejaculates collected from melatonin-treated rams was higher during week 9 compared to pre-implantation measures (4643.60 ± 56.44 vs 4331.09 ± 112 × 10^6^ spermatozoa/mL, respectively, p < 0.05) and was lower during week 30 compared to pre-implantation (3885.15 ± 234.10 vs 4331.09 ± 112.12 × 10^6^ spermatozoa/mL, respectively; p < 0.05). The sperm concentration of ejaculates collected from the control rams did not differ across the study period in comparison to study week 0 (P > 0.05).

**Figure 2:**
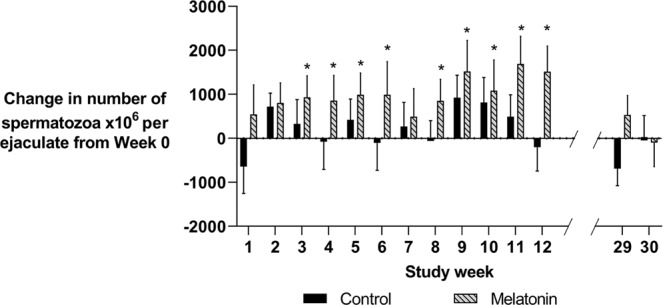
The difference in the number of spermatozoa × 10^6^ per ejaculate compared to week 0 measures for implanted and control**-**) Merino and Poll Dorset rams from study week 1 (1 week post-implantation) to study week 30 (following breeding season). Weekly values are presented as means ± S.E.M. Data is based off the 18 rams that collected from week 0 (Melatonin n = 9, Control n = 9). *Indicates significant difference from week 0 within treatment group (P < 0.05).

There was no effect of treatment or season upon ejaculate volume (P = 0.09), the overall average for which was 1.18 ml. However, both Merino and Poll Dorset melatonin -implanted rams increased the number of sperm per ejaculate compared to pre-implantation levels during study weeks 3–6 and 8–12 (P < 0.05, Fig. 2). There was no effect of melatonin on the number of sperm per ejaculate in the following breeding season (P > 0.05). Control rams did not differ from pre-implantation levels throughout the study period (P > 0.05).

There was no effect of melatonin observed upon ejaculate consistency score in either breed (P > 0.05, see Supplementary Fig. S3).

There was no effect of melatonin treatment on ejaculate wave motion or subjectively scored sperm motility (P > 0.05). Regardless of treatment group, all rams displayed higher wave motion scores during study weeks 9 and 29 (P < 0.05, see Supplementary Fig. S4). Compared to week 0, rams only displayed a greater percentage of sperm motility during week 9 (P < 0.001, see Supplementary Fig. S5), indicating a lack of seasonal distinction in sperm motility.

There was no effect of melatonin treatment or season on the percentage of total abnormal sperm (melatonin 6.60 ± 0.5%, control 11.19 ± 0.9%, P > 0.05). Regardless of treatment or study week, Poll Dorset rams had a higher average percentage of abnormal sperm than Merino rams (14.6 ± 1.0% vs 4.3 ± 0.3% respectively, P = 0.024).

**Figure 3:**
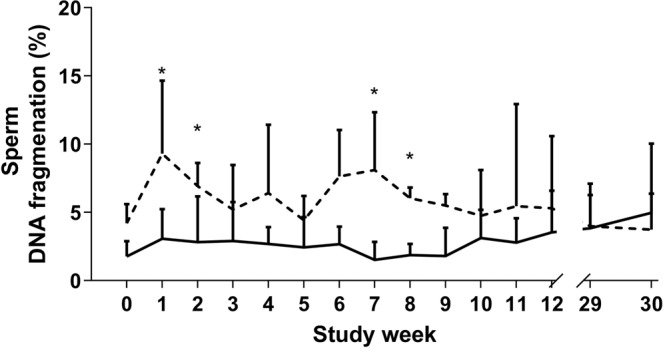
The percentage of sperm DNA fragmentation of melatonin implanted (-) and control (**-**) Poll Dorset rams (n = 8) from study week 0 (prior to implantation) to study week 30 (following breeding season). Weekly values are presented as means ± S.E.M. *Indicates significant difference between treatment groups (P < 0.05).

Differences in sperm DNA integrity between treatment groups were observed only in Poll Dorset rams, where control Poll Dorset rams showed higher average DNA fragmentation during study weeks 1–2,7–8 compared to melatonin-treated Poll Dorset rams (P < 0.05, Fig. 3). There was no effect of melatonin upon sperm DNA integrity from Merino rams.

### Melatonin modifies ram reproductive endocrinology

**Figure 4:**
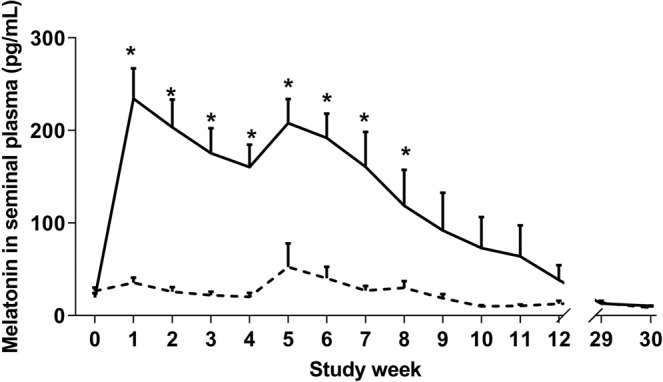
Seminal plasma concentrations of melatonin in implanted (-) and control (**-**) rams (n = 31) from study week 0 (prior to implantation) to study week 30 (following breeding season). Weekly values are presented as means ± S.E.M. *Indicates significant difference between treatment groups (P < 0.05).

Melatonin concentration in the seminal plasma of all melatonin- treated rams was higher (P < 0.001) compared to control rams from study weeks 1–8 (Fig. 4). No difference between control and treatment was observed in the pre-implantation period, study weeks 9–12, nor in the following breeding season (P > 0.05).

**Figure 5:**
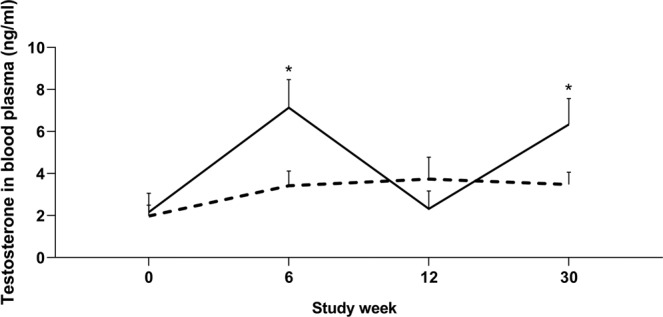
Blood plasma concentrations of testosterone in implanted (-) and control (**-**) rams (n = 31) at study week 0 (prior to implantation), 6, 12 and 30 (following breeding season). Weekly values are presented as means ± S.E.M. *Indicates significant difference between treatment groups (P < 0.05).

Melatonin concentrations in blood plasma (Fig. 5) were higher in both breeds of melatonin-implanted rams at study week 6 compared to control rams (266.70 ± 0.5 pg/mL vs 4.28 ± 0.7 pg/mL respectively, P < 0.001). There was no difference between melatonin and control rams during week 0 (8.96 ± 0.7 pg/mL vs 5.06 ± 1.4 pg/mL respectively, P > 0.05), week 12 (29.60 ± 16.6 vs 2.87 ± 0.9 respectively, P > 0.05) or week 30 (8.78 ± 0.8 vs 3.30 ± 0.7 respectively, P > 0.05).

Prior to melatonin implantation (study week 0), no difference in serum testosterone concentrations was observed between treatment groups. All melatonin-treated rams had elevated testosterone concentrations at study week 6 and in the following breeding season at study week 30 in comparison to both controls and pre-implantation levels (P < 0.005, Fig. 5). Control rams demonstrated no change in serum testosterone levels throughout the study (P > 0.05).

There was no effect of treatment on concentration of AMH in seminal plasma throughout the study (P > 0.05, see Supplementary Fig. S6).

Regardless of study week or treatment, AMH concentration in seminal plasma was positively correlated with the number of sperm per ejaculate produced (*r* = 0.464, P < 0.001) and sperm motility (*r* = 0.3242, P < 0.001). The range of AMH concentration in ram seminal plasma was 1.55–325.30 ng/mL, with a median concentration of 91.14 ng/ml.

There was no effect of treatment on concentration of inhibin A in seminal plasma throughout the study (P > 0.05, see Supplementary Fig. S7). The concentration of inhibin A in seminal plasma was weakly correlated to the number of sperm per ejaculate (*r* = 0.1786, P = 0.0135). The range of inhibin A concentration in ram seminal plasma was 0.91–284.90 pg/mL, with median 35.78 pg/ml.

## Discussion

The effects of exogenous melatonin upon ram reproduction have remained ambiguous for several years, and as such this study is the first to observe an extensive range of reproductive parameters over a long-term period following melatonin implantation in the ram. Individually, previous reports indicate that melatonin is able to influence some aspects of ram reproductive seasonality, though the time period over which these changes occur, and whether they occur simultaneously, is not well defined. We found that melatonin implantation results in a substantial increase in ram scrotal circumference, and importantly, sperm production with no compromise to semen quality. This study verifies the ability of exogenous melatonin to advance the ram breeding season and increase testicular function. Furthermore, as melatonin concentration and sperm production exceeded that of the natural breeding season, our results indicate that melatonin has other roles in improving testicular function aside from merely transcribing photoperiod to signal the onset of the breeding season. This study supports that melatonin merits application in industry to improve the reproductive efficiency of rams and allow for flexibility in reproductive management.

We observed that melatonin was elevated in ram seminal plasma the week following implantation, and that these levels remained higher in melatonin-treated rams until 10 weeks post implantation. Despite findings that seminal plasma melatonin concentration peaks 8–9 weeks after treatment in Raga Aragonesa rams^34^ our highest concentrations were observed the week following implantation, supporting numerous reports of melatonin’s ability to rapidly infiltrate peripheral tissues^44^. Furthermore, we saw no difference between treatment groups from week 9 onwards, although reports suggest implants can release the hormone upwards of 12 weeks^34,45^. Given that the same dosage of melatonin was applied across studies, these differences may be due to inter- breed variation. Though not statistically significant, we found similarly varying patterns of seminal plasma melatonin over time between Merino and Poll Dorset breeds (data not shown). As the Poll Dorset is a highly seasonal breed, and the Merino is not, differences in treatment response are somewhat expected, as British breeds tend to demonstrate a greater reproductive regression during the non-breeding period^46,47^. Briefly, though melatonin-treated rams weighed slightly less during the final two study weeks of the non-breeding season, and were heavier during the following breeding season, this difference was very slight (within 3 kilograms of controls). Given that body condition score did not differ between treatment groups, this variation is likely biologically irrelevant to any effects of treatment.

Melatonin was substantially higher in ram blood plasma at 6 weeks post-implantation. These values occur in a similar timeframe and range to those previously reported in Mediterranean breeds^34^. Interestingly, values of both seminal and blood plasma melatonin were comparable between the non-breeding and breeding season values in control rams, in agreement with previous findings using exogenous melatonin^34^ but conflicting with reports of natural changes where seminal plasma melatonin was elevated in the breeding season^48,49^. Given that we observed an increase in some reproductive parameters in the breeding period, it could be hypothesised that initial changes in melatonin concentration occur, and assert an effect, at the localised level of the hypothalamus and pituitary. Collection of data during daylight hours may have also impacted this result, as melatonin concentrations are known to be elevated during hours of darkness^50^. As the regulation of ovine reproduction is thought to be primarily based upon the increasing pattern of melatonin exposure, rather than concentration, that precedes the shorter days of the breeding season^6,51^, it is interesting that such high exogenous concentrations seem to be required to artificially exert a physiological effect in the ram. This suggests, given that sperm production in treated rams exceeded the natural breeding season values, that melatonin has other direct roles in testicular function, which we discuss further below.

In the ram, testosterone is primarily secreted from Leydig cells in the testes and is positively correlated with libido, sperm production and quality^10,52,53^. Here, we found that plasma testosterone in melatonin-treated rams was elevated from baseline values at 6 weeks post-implantation, with levels decreased to that of control rams at 12 weeks post-implantation, coinciding with previous studies^34,54^. This increase in testosterone supports that melatonin induces ram reproductive seasonality in both seasonal and non-seasonal breeds. Interestingly, our study showed that melatonin-treated rams had higher serum testosterone in the breeding season compared to both their control counterparts and baseline measures. As far as we are aware, this has not previously been reported. It is possible that this effect can be at least partially attributed to a direct influence of melatonin upon Sertoli cells. results which then binds to IGF-1 receptors upon Leydig cells to promote testosterone secretion. Furthermore, melatonin acts via melatonin-receptor 1 (MT1) to reduce oestradiol secretion, a known inhibitor of androgen production in the male gonad^10,55^. In other tissue types, upregulation of melatonin receptors MT1 and MT3 is shown to occur following melatonin exposure^56,57^. Though this study did not evaluate oestradiol concentrations in seminal plasma, relatively low seminal plasma oestradiol to testosterone ratios in melatonin-treated rams has previously been described^34^. Oestradiol is normally present in the testes following conversion from testosterone by aromatase P450^58^, where it then forms part of the negative feedback loop within the hypothalamic-pituitary-gonadal axis to regulate testosterone production^59^. Melatonin is reported to inhibit aromatase activity through MT1 binding, subsequently decreasing the conversion of testosterone to estrogen^60–63^. Given that testosterone secretion from the testis appears to be directly influenced by MT1 binding^10,55,64^, it is possible that exogenous melatonin in the ram upregulates the expression of melatonin receptors, which results in increased testosterone secretion, and reduces conversion of testosterone to oestrogen. Therefore, melatonin-treated rams may produce more testosterone in the subsequent breeding period compared to rams that have not been exposed to exogenous melatonin.

Another emerging marker of testicular function, AMH is produced by Sertoli cells, reaching a peak around puberty and retaining paracrine control of testicular function over the lifespan of the individual^39,40^. As an identifying marker for Sertoli cell function, seminal plasma levels of AMH are correlated to improved semen production and quality in humans^39–43^, and to sperm concentration in the boar^65^. Whilst increases in seminal plasma AMH in melatonin-treated rams failed to reach significance, this study supports that AMH is related to Sertoli cell function in the ram. We found a positive relationship between seminal plasma AMH and sperm production and sperm motility, despite one previous investigation in the ram suggesting that AMH in rete testis fluid has no correlation to Sertoli cell number^66^. In the ram, increasing concentration of inhibin A are thought to mark the transition into breeding season, where peak concentrations indicate that testis are fully active and functional^67^. Though seminal plasma Inhibin B is also similarly indicative of testicular function in other species^36,37,68^, we found only a weak relationship between inhibin A in seminal plasma and sperm motility, and did not identify any other clear relationship to treatment or sperm parameters.

Findings concerning the effect of exogenous melatonin upon ovine sperm production and quality are widely varied. Some studies note improvements in testicular size^69^, sperm motility^29,33,69^ and concentration^69^. On the contrary, these parameters have also exhibited no corresponding change to melatonin treatment^31,70–72^. This study aimed to clarify these discrepancies by utilising a relatively large sample size and biweekly measurements over a long-term period. In melatonin-implanted rams, we found increases in testicular size and sperm production, but no changes in sperm motility or morphology. Testicular size and the number of sperm per ejaculate were both elevated from 3 weeks post-implantation, suggesting a proliferation of testicular parenchyma as occurs in the natural breeding season. Past examinations of gonadotroph secretion patterns following melatonin treatment suggest that a down regulation of prolactin occurs, promoting an upregulation of follicle stimulating hormone (FSH) and luteinising hormone (LH)^3,35,73^. Combined with elevated testosterone secretion following melatonin implantation, the upregulation of these endocrine factors likely contributes to the proliferation of functional cells and promotes spermatogenesis.

As an *in vitro* additive, melatonin is demonstrated to improve sperm quality across species^12,18,20,74–76^, where changes are primarily attributed to its antioxidant properties. Investigated to a far lesser extent, the effect of exogenous melatonin upon sperm quality in the ram remains debated^28,33,70,77^. The present study demonstrated no effects of melatonin upon sperm motility or morphology throughout the entirety of the study, despite observing high levels of the hormone in the seminal plasma of melatonin-treated rams. Interestingly, there was little difference in sperm motility or morphology between the breeding and non-breeding seasons, suggesting these parameters may not be as greatly influenced by season in these breeds. We did observe differences in the percentage of sperm DNA fragmentation between melatonin and control Poll Dorset rams. Though melatonin and control Poll Dorset rams showed no difference at study week 0, control Poll Dorset rams had significantly higher levels of sperm DNA fragmentation during the several weeks of the non-breeding season compared to their melatonin-treated counterparts. However, as the melatonin group did not display any improvement following implantation, but rather retained consistently low fragmentation, we cannot conclusively credit these differences to treatment. This lack of improvement in overall sperm quality could be attributed to the fact that many rams in the melatonin group coincidentally had high quality ejaculates prior to implantation. However, melatonin did not negatively impact sperm quality despite causing increases in sperm production. As such, melatonin-treated rams were able to increase sperm output whilst maintaining ejaculate quality.

This study found that exogenous melatonin increased the likelihood of rams producing an ejaculate over time, with the effect only seen in the more seasonal Poll Dorset breed at 9–12 weeks post-implantation. Though there was an increase in reproductive behaviours during the breeding season, the lack of treatment effect upon all other measures of libido may be due to the housing of the rams, where all rams were maintained in a single flock throughout the study. Rams are known to vary their sexual behaviour in response to different social environments, and when in the presence of competitors, which may influence social hierarchy^78–80^. It is possible that both the housing and semen collection environment, where all rams were in direct competition, influenced the ram libido at time of collection and masked any influence of treatment. Testing during the 6–9 weeks post-implantation, where higher concentrations of melatonin and testosterone were present, may have yielded clearer results on the effect of melatonin upon ram sexual behaviour.

In summary, previous investigations into the effects of exogenous melatonin have presented conflicting information as to whether this pineal neurohormone is able to truly induce reproductive seasonality in the ram during the non-breeding period. This study has clarified these effects in both a seasonal and non-seasonal breed, supporting the ability of exogenous melatonin to advance the ram breeding season and improve sperm production. The findings of this study justify the use of melatonin in industry to increase ram reproductive potential, allowing for increased productivity and flexibility of reproductive management.

## Methods

### Animals

This study was carried out in strict accordance with the Australian Research Act 1985 No. 123 and the Australian code for the care and use of animals for scientific purposes 8^th^ edition (2013).

All experimental procedures were conducted with approval from the University of Sydney Animal Ethics Committee (approval 2017/1155). All animals were housed at the University of Sydney Sheep Research Unit, Cobbitty NSW, Australia. Poll Dorset (n = 14) and Merino (n = 17) rams were maintained as a single mob on pasture. Rams had access to improved pasture (couch, kikuyu, kangaroo grass, rye, broome, oat, triticale) and water ad libitum. Additional lucerne and lupins were supplemented up to three times a week.

Rams were selected for trial inclusion conditional to veterinary observation of normal reproductive parameters and health, with animals excluded from the study if testicular lesions, disease, low body condition score or high temperatures were observed. All animals were approximately 2 years old. Ram health was assessed by a veterinarian approximately every 6 weeks throughout the study, including assessment of blood biochemistry and hematology, respiratory and heart rate, body condition score, body temperature and visual observation of any external wounds or lesions (data not presented).

Teaser ewes (n = 20) involved in semen collection and libido testing procedures were housed as a single flock on improved pasture with access to water ad libitum. 4 ewes were synchronised for estrus approximately 2 weeks prior to each semen collection or libido testing event, using industry standard methods^81^.

### Experimental design

The study was conducted under VICH Good Clinical Practice (GCP) guidelines (International Co-operation on Harmonisation of Technical Requirements for Registration of Veterinary Medicinal Products, Good Clinical Practice (GL9), June 2000, effective July 2001.

The study was a 2 × 2 factorial experimental design, where rams were randomly allocated into melatonin and control groups, with an even distribution of weight and breed. With the exception of the primary investigator, all other investigators were blinded to treatment allocation to prevent bias in subjective measures. All rams were trained for semen collection 1 month prior to initiation of the study using teaser ewes and an artificial vagina. Following the initial two weeks of baseline data collection, where no animals had received melatonin, approximately half the rams in each breed (Merino + melatonin n = 8; Merino control n = 9; Poll Dorset + melatonin n = 7; Poll Dorset control n = 7) received three 18 mg subcutaneous melatonin implants (Regulin, Ceva Animal Health, Australia) behind their left ear. Control rams underwent the same implantation process with empty implanter guns.

Ram physical and behavioural data and duplicate semen samples were collected weekly over 14 weeks during the Australian non-breeding season (September-December) and 2 weeks in the following breeding season (April). During each week, two semen samples per ram were collected on the same day, approximately 3 hours apart. The study timeframe was based on evidence that implants release melatonin over an approximated 80 day period, with high serum and seminal plasma levels expected in the initial 8 weeks^34^.

All equipment used was calibrated weekly prior to measurements. Unless otherwise stated all chemicals were supplied by Sigma-Aldrich (St Louis, MO, USA).

All subjective measurements were performed consistently by a trained individual throughout the study period.

### Ram libido, scrotal circumference, body weight and condition score

#### Scrotal circumference

Scrotal circumference was measured each week the day prior to semen collection by pushing the testes ventrally and placing livestock testes measuring tape at the widest anteroposterior diameter of the scrotum.

#### Ram libido

During weekly semen collection, ram libido was subjectively scored out of 4 (1: disinterested, no attempt to mount, 4: highly interested in ewe, mounting attempts, courting and flehmen displayed). Time taken to ejaculate also contributed to libido score. Scoring was performed by a single individual throughout the study. Whether a ram ejaculated or not was recorded for all semen collections.

Advanced libido testing was performed in at the end of the non- breeding season (study week 13) and in the following breeding season (study week 31). During testing, individual rams were placed in a pen with a single synchronized ewe and the number of ram behaviours (sniffs, flehmen response, courting, attempted mounting and ejaculations) performed were recorded over a 20 minute period. All rams were tested in the same testing environments and conditions. Libido testing pens were situated away from other sheep, with two observers per pen hidden from view. The test was replicated twice in both the breeding and non-breeding periods.

#### Weight and body condition score

At the same time and day each study week, rams were weighed in a calibrated walk-over weigh system (Gallagher Sheep Auto drafter, model G05714) following body condition scoring as per industry standard^82^.

### Semen production and quality

#### Semen collection

Prior to study initiation, rams were trained to ejaculate into an artificial vagina in the presence of a synchronised teaser ewe. Using this method, two ejaculates were collected from each ram once per week throughout the duration of the study. Time taken to ejaculate was recorded, with no collection if the ram did not ejaculate within 4 minutes.

#### Semen volume, concentration and consistency

Immediately following collection, semen volume was observed by measuring the weight of the total ejaculate to the nearest 0.01 g. Concentration of the raw ejaculate was determined by averaging three readings on a calibrated colorimeter (Photometer SDM1, Minitube, Victoria Australia). Measurements of volume and concentration were then used to calculate the number of sperm per ejaculate.

Each ejaculate was subjectively scored for consistency (the ratio of sperm to seminal plasma) out of 5^81^.

#### Wave motion and motility

Ejaculate wave motion was determined at 100× magnification as per industry protocol^81^. Subjective motility was observed by diluting a portion of each ejaculate to a concentration of 100 × 10^6^ spermatozoa/mL with Salamon’s diluent for fresh/chilled semen (300 mM Tris, 104 mM citric acid, 28 mM fructose, 15% v/v egg yolk, pH 7.3) and observing at 200× magnification.

#### Sperm morphology

Morphology slides for each ejaculate were prepared by diluting samples in phosphate-buffered saline shortly after ejaculation to a concentration of 100 × 10^6^ spermatozoa/ml.

The number of morphological abnormalities were subjectively determined per 200 cells for each ejaculate as per industry standard^81^.

#### Sperm DNA integrity

Immediately after semen collection, 20 ul of each ejaculate was snap frozen by submerging in liquid nitrogen for 30 seconds and storing at −80 °C until assessment.

DNA integrity was assessed by flow cytometry (C6 BD Accuri, Becton Dickinson, New Jersey, USA) using acridine orange staining as similarly described by Evenson *et al*., 2000^83^, with some adjustments. Briefly, samples were diluted to a concentration of 1–2 × 10^6^ spermatozoa/mL in 200 ul with 1× TNE buffer (0.15 M NaCl, 0.01 M Tris HCl, 1 mM disodium EDTA pH 7.4). Samples were then diluted with 400 ul acid detergent solution (0.08NHCl, 0.15 M NaCl, 0.1% Triton × 100 pH 1.2). Exactly 30 seconds later, samples were stained with 1.2 ml acridine orange (6 ug/ml). Stained samples were incubated for 3 minutes before assessment by flow cytometry, where green fluorescence (FL1) was detected using 533/30 band pass filter, and red fluorescence (FL3) a 670 long pass filter. Flow rate was set to 200 events per second, with a minimum of 5000 sperm cells recorded per sample.

DNA fragmentation was estimated by the relative amount of single stranded and double stranded DNA, indicated by the proportion of sperm demonstrating red fluorescence (i.e. red fluorescence/red + green fluorescence).

### Seminal and blood plasma hormone analysis

#### Seminal and blood plasma extraction

Immediately after initial evaluation, the remainder of each ejaculate was centrifuged twice at 14,000 g for 20 minutes, with aspiration of the supernatant following each centrifugation in order to isolate seminal plasma and remove sperm cells. Seminal plasma was stored at −80 °C prior to analysis.

5–10 mL of peripheral blood was collected from the jugular vein of each ram during study weeks 0, 6, 12 and 30 the day prior to semen collection. To separate plasma from cells, samples were treated with heparin and centrifuged at 3500 × g for 20 mins. Plasma was stored at −80 °C prior to analysis.

#### Melatonin

Melatonin levels in seminal and blood plasma were determined by reverse-phase C-18 column extraction of plasma or seminal fluid samples, followed by double antibody radioimmunoassay (RKMEL-2, Buhlmann Laboratories AG, Schönenbuch, Switzerland) at the Adelaide Research Assay Facility. This assay is based on the Kennaway G280 anti-melatonin antibody^84^ and uses ^[125I]^2-iodomelatonin as the radioligand.

250 µl aliquots of plasma or seminal fluid were extracted using reverse-phase C-18 columns and then resuspended in 1000 µl of supplied assay buffer, resulting in a 4-fold dilution. Control samples of plasma or seminal fluid were assayed at this dilution step, while samples from melatonin-treated rams were diluted a further 5-fold in assay buffer and assayed at a 20-fold dilution step. The extra dilution step is required to keep all samples within the standard curve range.

For the control and melatonin group samples, assay sensitivity was 2.0 pg/mL and 10 pg/mL respectively. Intra-assay CV was 6.6% and inter-assay CV was 12.1%.

#### Testosterone

Testosterone in blood plasma was quantified in duplicate using Mini-Vidas automated enzyme linked fluorescent assay (bioMérieux, NSW Australia) as per manufacturer’s instructions. Assay sensitivity was 0.1 ng/ml. The Vidas system has been previously validated against established assays for detected of testosterone^85,86^.

#### Anti Mullerian hormone (AMH)

Anti-Mullerian hormone concentrations were determined using Ovine AMH quantitative three-step sandwich ELISA kits (Ansh Labs LLC, Webster, TX) following manufacturer protocol. Briefly, 50 μL of calibrators, controls and seminal plasma samples were added in duplicate to AMH antibody coated microtiter wells. 50 ul AMH assay buffer premix was added to wells and incubated for 2 hours. Following initial incubation and washing, wells were incubated with 100 μl biotinylated AMH antibody for 1 hour. After this second incubation and washing, wells were incubated with 100 μL streptavidin-horseradish peroxidase conjugate for 30 mins. After the third incubation and washing, wells were incubated with 100 ml chromogenic substrate (TMB) for 9 minutes. Following the addition of stopping solution (sulfuric acid), and absorbance measured on a microtiter plate reader (TS800 microplate reader, Biotek) at 450 nm with background wavelength correction at 630 nm. Assay sensitivity was 0.025 ng/ml and intra and inter-assay variability was 9.93% and 4.95% respectively.

As only 18 of the 31 rams (Merino + melatonin n = 5; Merino control n = 5; Poll Dorset + melatonin n = 4; Poll Dorset control n = 4) produced ejaculates in the study weeks prior to implantation, only seminal plasma samples from this subset of rams was analysed in order to accurately compare later study weeks to the pre-implantation period.

#### Inhibin A

Inhibin-A concentrations in seminal plasma were determined using Inhibin A quantitative three-step sandwich ELISA kits (Ansh Labs LLC, Webster, TX) following manufacturer protocol. Briefly, 50 μl of calibrators, controls and seminal plasma samples were added in duplicate to Inhibin A antibody coated microtiter wells. 50 ul Inhibin A assay buffer premix was added to wells and incubated for 2.5 hours. Following initial incubation and washing, wells were incubated with 100 μl biotinylated inhibin α-subunit antibody for 1 hour. After this second incubation and washing, wells were incubated with 100 μl streptavidin-horseradish peroxidase conjugate for 30 mins. After the third incubation and washing, wells were incubated with 100 μl chromogenic substrate (TMB) for 12 minutes. Following the addition of stopping solution (sulfuric acid) and absorbance measured on a microtiter plate reader (TS800 microplate reader, Biotek) at 450 nm with background wavelength correction at 630 nm. Assay sensitivity was 2.3 pg/ml and intra and inter-assay variability was 7.8% and 11.32% respectively.

As with AMH analysis (2.4.4), only the subset of 18 rams collecting during pre-implantation weeks were analysed for seminal plasma inhibin A concentration.

### Statistical analysis

The number of sperm per ejaculate, motility, DNA integrity and hormone concentrations were statistically analysed using linear mixed model regression (REML) in R 3.4.1^87,88^. For the layout of these models, study week and breed were set as fixed effects and ram and ejaculate were set as random effects. Normality and homogeneity of residual variances were confirmed using the Shapiro-Wilk test and Bartlett’s test, respectively. If required, the data were transformed to correct for unequal variances. Manipulation of the model to reduce heteroscedasticity of the residuals was performed if necessary and, in some cases, this required the application of a log transformation. If a log transformation was performed, the results were back transformed and presented as the geometric mean ± 95% confidence intervals.

All ordinal data (wave motion, ejaculate colour, libido) were analysed using ordinal logistic regression in R 3.4.1. using ‘clmm’ within the ‘ordinal package’^89^. Statistical significance was considered if P < 0.05 and all values are reported as the mean ± standard error of the mean.

Data were compared between treatment groups on a per week basis, as well as within treatment group across the study period in order to determine any changes following melatonin implantation.

As only 18 of the 31 rams produced ejaculates in the study weeks prior to implantation, any statistical analysis comparing later study weeks to the pre-implantation period were based on this subset of rams (Merino + melatonin n = 5; Merino control n = 5; Poll Dorset + melatonin n = 4; Poll Dorset control n = 4). This was done to improve the accuracy of weekly comparisons, and in cases where within-week comparisons between treatment groups were not relevant. Data assessed using this method included sperm production and quality parameters, and AMH and Inhibin A concentrations in seminal plasma.

Correlations between AMH, Inhibin A and semen parameters were investigated by assessing associations using linear regression. If significant (P < 0.05), Pearson’s correlation test was then used to determine correlations. Correlations were considered significant if P < 0.05.

## Supplementary information

Supplementary Figure S1

Supplementary Figure S2

Supplementary Figure S3

Supplementary Figure S4

Supplementary Figure S5

Supplementary Figure S6

Supplementary Figure S7

Supplementary information

## References

[1] Malpaux B (1996). Seasonal breeding in sheep: Mechanism of action of melatonin. Anim. Reprod. Sci..

[2] Heiliwell RJA, Williams LM (1992). Melatonin Binding Sites in the Ovine Brain and Pituitary: Characterization During the Oestrous Cycle. J. Neuroendocrinol..

[3] Castle-Miller J, Bates DO, Tortonese DJ (2017). Mechanisms regulating angiogenesis underlie seasonal control of pituitary function. Proc. Natl. Acad. Sci..

[4] Fink, G., Pfaff, D. & Levine, J. Handbook of Neuroendocrinology. Handbook of Neuroendocrinology, 10.1016/C2009-0-04284-6 (2012).

[5] Lincoln, G. A. & Clarke, I. J. Photoperiodically‐lnduced Cycles in the Secretion of Prolactin in Hypothalamo‐Pituitary Disconnected Rams: Evidence for Translation of the Melatonin Signal in the Pituitary Gland. *J. Neuroendocrinol*., 10.1111/j.1365-2826.1994.tb00580.x (1994). 10.1111/j.1365-2826.1994.tb00580.x 7920591

[6] Bittman EL, Dempsey RJ, Karsch FJ (1983). Pineal melatonin secretion drives the reproductive response to daylength in the ewe. Endocrinology.

[7] Karsch FJ, Goodman RL, Legan SJ (1980). Feedback basis of seasonal breeding: test of an hypothesis. Reproduction.

[8] Lincoln GA (1990). Seasonal cycles in the blood plasma concentration of FSH, inhibin and testosterone, and testicular size in rams of wild, feral and domesticated breeds of sheep. J Reprod Fertil..

[9] Langford, G. A., Ainsworth, L., Marcus, G. J. & Shrestha, J. N. B. Photoperiod Entrainment of Testosterone, Luteinizing Hormone, Follicle-Stimulating Hormone, and Prolactin Cycles in Rams in Relation to Testis Size and Semen Quality1. *Biol. Reprod*., 10.1095/biolreprod37.2.489 (1987). 10.1095/biolreprod37.2.489 3118978

[10] Deng S-L (2018). Melatonin promotes sheep Leydig cell testosterone secretion in a co-culture with Sertoli cells. Theriogenology.

[11] González-Arto M (2017). Melatonin MT1 and MT2 receptors in the ram reproductive tract. Int. J. Mol. Sci..

[12] Gonzalez-Arto M (2016). New evidence of melatonin receptor contribution to ram sperm functionality. Reprod. Fertil. Dev..

[13] Casao A (2012). Identification and immunolocalisation of melatonin MT1 and MT2 receptors in Rasa Aragonesa ram spermatozoa. Reprod. Fertil. Dev..

[14] Gonzalez-Arto M (2014). New evidence of melatonin receptor contribution to ram sperm functionality. Reprod. Fertil. Dev..

[15] Casao A (2010). Seasonal variations of melatonin in ram seminal plasma are correlated to those of testosterone and antioxidant enzymes. Reprod. Biol. Endocrinol..

[16] Reiter RJ (2016). Melatonin as an antioxidant: under promises but over delivers. Journal of Pineal Research.

[17] Appiah MO, He B, Lu W, Wang J (2019). Antioxidative effect of melatonin on cryopreserved chicken semen. Cryobiology.

[18] Pang Y-W (2016). Protective effects of melatonin on bovine sperm characteristics and subsequent *in vitro* embryo development. Mol. Reprod. Dev..

[19] Karimfar M (2015). The protective effects of melatonin against cryopreservation-induced oxidative stress in human sperm. Int. J. Immunopathol. Pharmacol..

[20] Succu S (2011). Melatonin protects ram spermatozoa from cryopreservation injuries in a dose-dependent manner. J. Pineal Res..

[21] Cevik M, Yilmazer C, Kocyigit A (2017). Effects of melatonin implantation on the fertility potentials of Kivircik and Charollais ewes and rams during the non-breeding season. Pol. J. Vet. Sci..

[22] Luridiana S (2015). Melatonin treatment in spring and reproductive recovery in sheep with different body condition score and age. Anim. Reprod. Sci..

[23] Abecia, J. A., Palacín, I., Forcada, F. & Valares, J. A. The effect of melatonin treatment on the ovarian response of ewes to the ram effect. *Domest. Anim. Endocrinol*., 10.1016/j.domaniend.2005.09.003 (2006). 10.1016/j.domaniend.2005.09.003 16221539

[24] Zúñiga O, Forcada F, Abecia JA (2002). The effect of melatonin implants on the response to the male effect and on the subsequent cyclicity of Rasa Aragonesa ewes implanted in April. Anim. Reprod. Sci..

[25] Stellflug JN, Fitzgerald JA, Parker CF, Bolt D (1988). Influence of concentration, duration and route of administration of melatonin on reproductive performance of spring-mated polypay and polypay-cross ewes. J. Anim. Sci..

[26] Staples LD, McPhee S, Kennaway DJ, Williams AH (1990). The influence of exogenous melatonin on the seasonal patterns of ovulation and oestrus in sheep. Anim. Reprod. Sci..

[27] Haresign W, Peters AR, Staples LD (1990). The effect of melatonin implants on breeding activity and litter size in commercial sheep flocks in the UK. Anim. Prod..

[28] Rekik M (2015). Melatonin administration enhances the reproductive capacity of young rams under a southern Mediterranean environment. Anim. Sci. J..

[29] Kaya A, Baspinar N, Yildiz C, Kurtoglu F, Ataman MB, Haliloglu. (2000). Influence of melatonin implantation on sperm quality, biochemical composition of the seminal plasma and plasma testosterone levels in rams. Rev. Med. Vet. (Toulouse)..

[30] Rosa H, Juniper D, Bryant M (2000). Effects of recent sexual experience and melatonin treatment of rams on plasma testosterone concentration, sexual behaviour and ability to induce ovulation in seasonally anoestrous ewes. J. Reprod. Fertil..

[31] Palacín I (2008). Effects of exogenous melatonin treatment on out-of-season ram fertility. Ital. J. Anim. Sci..

[32] Buffoni A (2015). Melatonin modifies scrotal circumference but not plasma testosterone concentrations and semen quality of rams during the seasonal anestrus at 43°S. Biol. Rhythm Res..

[33] Casao A (2010). Effects of melatonin implants during non-breeding season on sperm motility and reproductive parameters in rasa aragonesa rams. Reprod. Domest. Anim..

[34] Casao A (2013). The effect of exogenous melatonin during the non-reproductive season on the seminal plasma hormonal profile and the antioxidant defence system of Rasa Aragonesa rams. Anim. Reprod. Sci..

[35] Lincoln, G. A. & Ebling, F. J. P. Effect of constant-release implants of melatonin on seasonal cycles in reproduction, prolactin secretion and moulting in rams. *J. Reprod. Fertil*., 10.1530/jrf.0.0730241 (1985). 10.1530/jrf.0.0730241 3918164

[36] Semercioz, A., Avunduk, A K Baltaci, C. & MogulkocMustafa, R. Effect of Zinc and Melatonin on Oxidative Stress and Serum Inhibin-B Levels in a Rat Testicular Torsion–Detorsion Model. *Biochem. Genet*., 10.1007/s10528-017-9826-5 (2017). 10.1007/s10528-017-9826-5 29094225

[37] Pierik FH, Vreeburg JTM, Stijnen T, De Jong FH, Weber RFA (1998). Serum inhibin B as a marker of spermatogenesis. J. Clin. Endocrinol. Metab..

[38] Mahmoud, A. M. Protective Effects of Umbelliferone in Experimental Testicular Ischaemia/Reperfusion Injury in Rats. *Anat. Physiol*. **6** (2015).

[39] Nery SF (2014). Seminal plasma concentrations of Anti-Müllerian hormone and inhibin B predict motile sperm recovery from cryopreserved semen in asthenozoospermic men: a prospective cohort study. Andrology.

[40] Marca L (2010). a *et al*. Anti-Mullerian hormone (AMH) as a predictive marker in assisted reproductive technology (ART). Hum. Reprod. Update.

[41] Fujisawa M, Yamasaki T, Okada H, Kamidono S (2002). The significance of anti-Mullerian hormone concentration in seminal plasma for spermatogenesis. Hum. Reprod..

[42] Mostafa T (2007). Seminal plasma anti-Müllerian hormone level correlates with semen parameters but does not predict success of testicular sperm extraction (TESE). Asian J. Androl..

[43] Duvilla E (2008). Significance of inhibin B and anti-Müllerian hormone in seminal plasma: a preliminary study. Fertil. Steril..

[44] Tordjman S (2017). Melatonin: Pharmacology, Functions and Therapeutic Benefits. Curr. Neuropharmacol..

[45] Satta V (2018). Effects of melatonin administration on seminal plasma metabolites and sperm fertilization competence during the non-reproductive season in ram. Theriogenology.

[46] Boland MP (1985). The influence of breed, season and photoperiod on semen characteristics, testicular size, libido and plasma hormone concentrations in rams. Anim. Reprod. Sci..

[47] Dacheux, J. L., Pisselet, C., Blanc, M. R., Hochereau-de-Reviers, M.-T. & Courot, M. Seasonal variations in rete testis fluid secretion and sperm production in different breeds of ram. *Reproduction*, 10.1530/jrf.0.0610363 (1981). 10.1530/jrf.0.0610363 7205782

[48] Carvajal-Serna M (2019). Changes in melatonin concentrations in seminal plasma are not correlated with testosterone or antioxidant enzyme activity when rams are located in areas with an equatorial photoperiod. Anim. Reprod. Sci..

[49] Casao, A. *et al*. variations of melatonin in ram seminal plasma are correlated to those of testosterone and antioxidant enzymes. *Reprod. Biol. Endocrinol*., 10.1186/1477-7827-8-59 (2010). 10.1186/1477-7827-8-59 PMC2907381 20540737

[50] Cardinali DP, Pévet P (1998). Basic aspects of melatonin action. Sleep Med. Rev..

[51] Karsch FJ (1986). Melatonin and Photorefractoriness: Loss of Response to the Melatonin Signal Leads to Seasonal Reproductive Transitions in the Ewe. Biol. Reprod..

[52] Belkadi S (2017). Seasonal influence on sperm parameters, scrotal measurements, and serum testosterone in Ouled Djellal breed rams in Algeria. Vet. World.

[53] Fernandez-Abella D, Becu-Villalobos D, Lacau-Mengido IM, Villegas N, Bentancur O (1999). Sperm production, testicular size, serum gonadotropins and testosterone levels in Merino and Corriedale breeds. in. Reproduction Nutrition Development.

[54] Kokolis N (2000). The effect of melatonin implants on blood testosterone and acrosin activity in spermatozoa of the ram. Andrologia.

[55] Carreau, S. Germ cells: A new source of estrogens in the male gonad. in Molecular and Cellular Endocrinology **178**, 65–72 (Elsevier, 2001). 10.1016/s0303-7207(01)00411-7 11403896

[56] Carbajo-Pescador S (2009). Changes in the expression of melatonin receptors induced by melatonin treatment in hepatocarcinoma HepG2 cells. J. Pineal Res..

[57] Masana MI, Witt-Enderby PA, Dubocovich ML (2003). Melatonin differentially modulates the expression and function of the hMT1 and hMT2 melatonin receptors upon prolonged withdrawal. Biochem. Pharmacol..

[58] Rochira, V. *et al*. Estrogens and male reproduction. (2016).

[59] Carreau S (2003). Aromatase expression and role of estrogens in male gonad: a review. Reprod. Biol. Endocrinol..

[60] Alvarez-García V, González A, Martínez-Campa C, Alonso-González C, Cos S (2013). Melatonin modulates aromatase activity and expression in endothelial cells. Oncol. Rep..

[61] González, A. *et al*. Effects of MT1 melatonin receptor overexpression on the aromatase-suppressive effect of melatonin in MCF-7 human breast cancer cells. *Oncol. Rep*. (2007). 10.3892/or.17.4.947 17342341

[62] Cos S, Martinez-Campa C, Mediavilla MD, Sanchez-Barcelo EJ (2005). Melatonin modulates aromatase activity in MCF-7 human breast cancer cells. J. Pineal Res..

[63] Cos S (2006). Estrogen-signaling pathway: A link between breast cancer and melatonin oncostatic actions. Cancer Detect. Prev..

[64] Gao Y (2019). Melatonin receptor depletion suppressed hCG-induced testosterone expression in mouse Leydig cells. Cell. Mol. Biol. Lett..

[65] Barranco I (2020). Seminal Plasma Anti-Müllerian Hormone: A Potential AI-Boar Fertility Biomarker?. Biology (Basel)..

[66] Cazorla, O. *et al*. Anti-Mullerian hormone (AMH) secretion in prepubertal and adult rams. *J. Reprod. Fertil*., 10.1530/jrf.0.1120259 (1998). 10.1530/jrf.0.1120259 9640265

[67] McNeilly AS (2002). Production of inhibin A not B in rams: Changes in plasma inhibin A during testis growth, and expression of inhibin/activin subunit mRNA and protein in adult testis. Reproduction.

[68] Von Eckardstein, S. *et al*. Serum inhibin B in combination with serum follicle-stimulating hormone (FSH) is a more sensitive marker than serum FSH alone for impaired spermatogenesis in men, but cannot predict the presence of sperm in testicular tissue samples. *J. Clin. Endocrinol. Metab*., 10.1210/jc.84.7.2496 (1999). 10.1210/jcem.84.7.5855 10404826

[69] Egerszegi I (2014). Effect of melatonin treatment on semen parameters and endocrine function in Black Racka rams out of the breeding season. Small Rumin. Res..

[70] Rosa HJD, Silva CC, Bryant MJ (2012). The effect of melatonin treatment in rams on seasonal variation of testicular size and semen production parameters. Small Rumin. Res..

[71] Faigl V (2009). Testicular function and semen characteristics of Awassi rams treated with melatonin out of the breeding season. Acta Vet. Hung..

[72] Sookhtehzari, A., Vojgani, M., Niassari Naslaji, A., Bahonar, A. & Gholami, G. Influence of melatonin treatmenton scrotalcircumference and semen parameters in shallrams on out of season. *J. Vet. Res*. (2009).

[73] Lincoln, G. A. & Clarke, l. J. Refractoriness to a Static Melatonin Signal Develops in the Pituitary Gland for the Control of Prolactin Secretion in the Ram. *Biol. Reprod*., 10.1095/biolreprod57.2.460 (1997). 10.1095/biolreprod57.2.460 9241064

[74] Casao BA (2010). Melatonin prevents capacitation and apoptotic-like changes of ram spermatozoa and increases fertility rate. J. Pineal Res..

[75] Espino J (2010). Melatonin as a potential tool against oxidative damage and apoptosis in ejaculated human spermatozoa. Fertil. Steril..

[76] Rao MV, Gangadharan B (2008). Antioxidative potential of melatonin against mercury induced intoxication in spermatozoa *in vitro*. Toxicol. Vitr..

[77] Ramadan TA, Taha TA, Samak MA, Hassan A (2009). Effectiveness of exposure to longday followed by melatonin treatment on semen characteristics of Damascus male goats during breeding and non-breeding seasons. Theriogenology.

[78] Ungerfeld R, Lacuesta L (2010). Social rank during pre-pubertal development and reproductive performance of adult rams. Anim. Reprod. Sci..

[79] Ungerfeld R, González-Pensado S (2009). Social Dominance and Courtship and Mating Behaviour in Rams in Non-Competitive and Competitive Pen Tests. Reprod. Domest. Anim..

[80] Patel M, Das N, Pandey HN, Yadav MC, Girish PS (2006). Ram Mating Behaviour under Different Social Conditions. *Asian-Australasian*. J. Anim. Sci..

[81] Evans, G. & Maxwell, W. M. C. Salamons’ artificial insemination of sheep and goats. *Salamons’**Artif. Insemin. sheep goats* (1987).

[82] Russel, A. Body condition scoring of sheep. *In Pract*, 10.1136/inpract.6.3.91 (1984). 10.1136/inpract.6.3.91 6735487

[83] Evenson D, Jost L (2000). Sperm chromatin structure assay is useful for fertility assessment. Methods Cell Sci..

[84] Kennaway DJ, Voultsios A (1998). Circadian Rhythm of Free Melatonin in Human Plasma. J. Clin. Endocrinol. Metab..

[85] Taieb J (2003). Testosterone Measured by 10 Immunoassays and by Isotope-Dilution Gas Chromatography–Mass Spectrometry in Sera from 116 Men, Women, and Children. Clin. Chem..

[86] Grasso L, Fragomeni F, Cecconi E, Gasperi M (2002). Comparison of VIDAS testosterone assay with chemiluminescence and RIA methods. Fertil. Steril..

[87] Bates, D., Mächler, M., Bolker, B. M. & Walker, S. C. Fitting linear mixed-effects models using lme4. *J. Stat. Softw*., 10.18637/jss.v067.i01 (2015).

[88] R Core Team. R: A language and environment for statistical computing. R Found. Stat. Comput. Vienna, Austria, http//www.R-project.org/ (2017).

[89] Christensen, R. Package Ordinal: Regression Models for Ordinal Data. R Packag. version 2015 (2015).

