# Supplementary figures and images for: Exogenous melatonin advances the ram breeding season and increases testicular function
Source: Sci Rep. 2020 Jun 16;10:9711. doi: 10.1038/s41598-020-66594-6 (PMC7297710; doi:10.1038/s41598-020-66594-6)

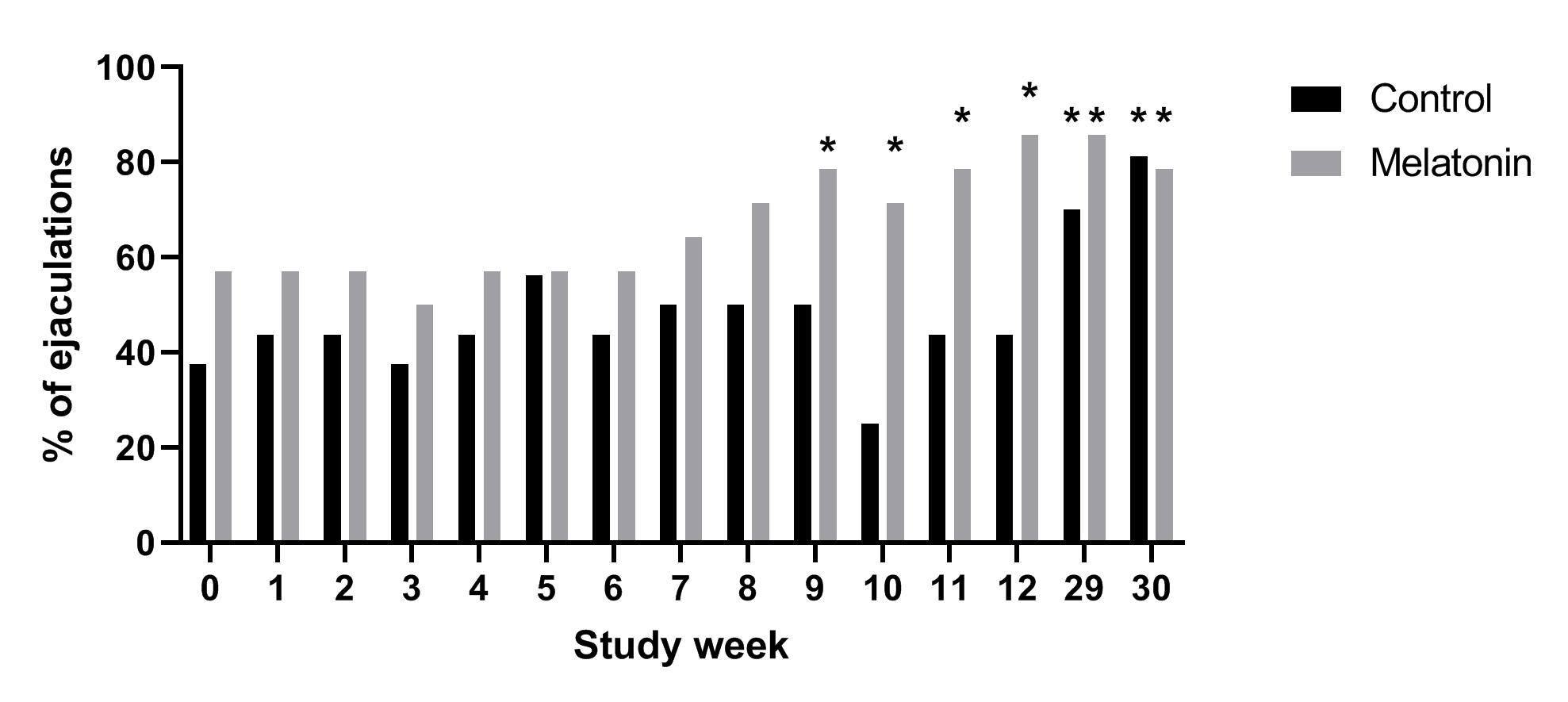

Supplement: Supplementary file 1 — Supplementary Figure S1 [file 41598_2020_66594_MOESM1_ESM.jpg]

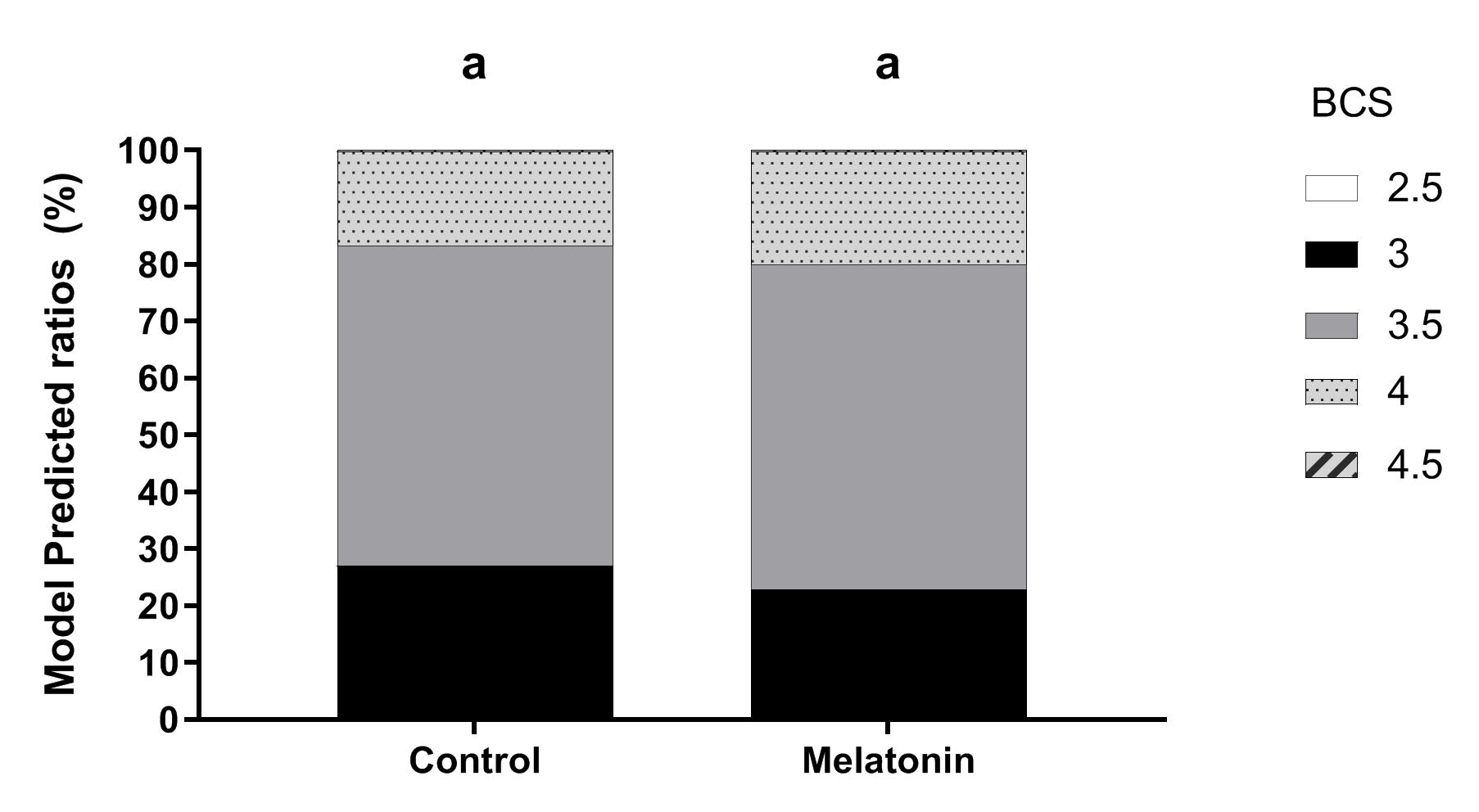

Supplement: Supplementary file 2 — Supplementary Figure S2 [file 41598_2020_66594_MOESM2_ESM.jpg]

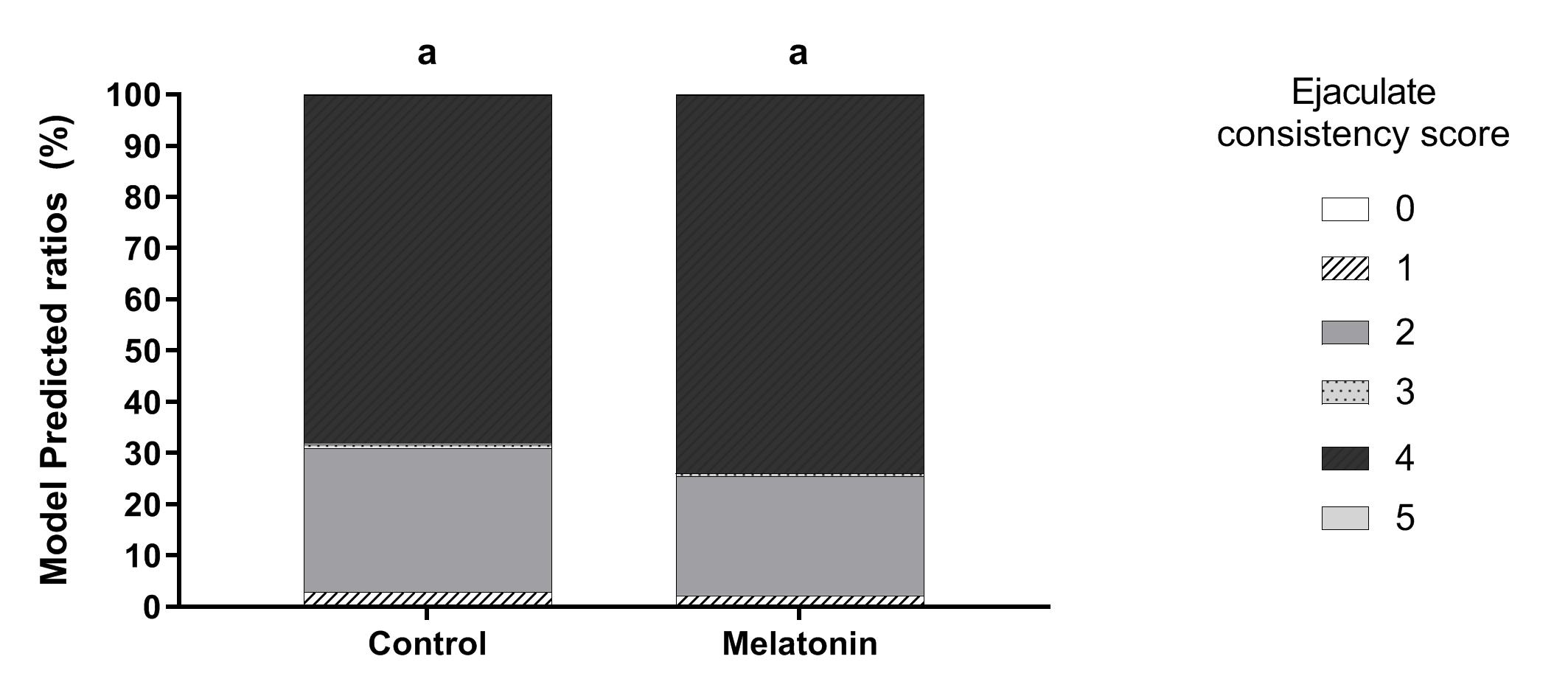

Supplement: Supplementary file 3 — Supplementary Figure S3 [file 41598_2020_66594_MOESM3_ESM.jpg]

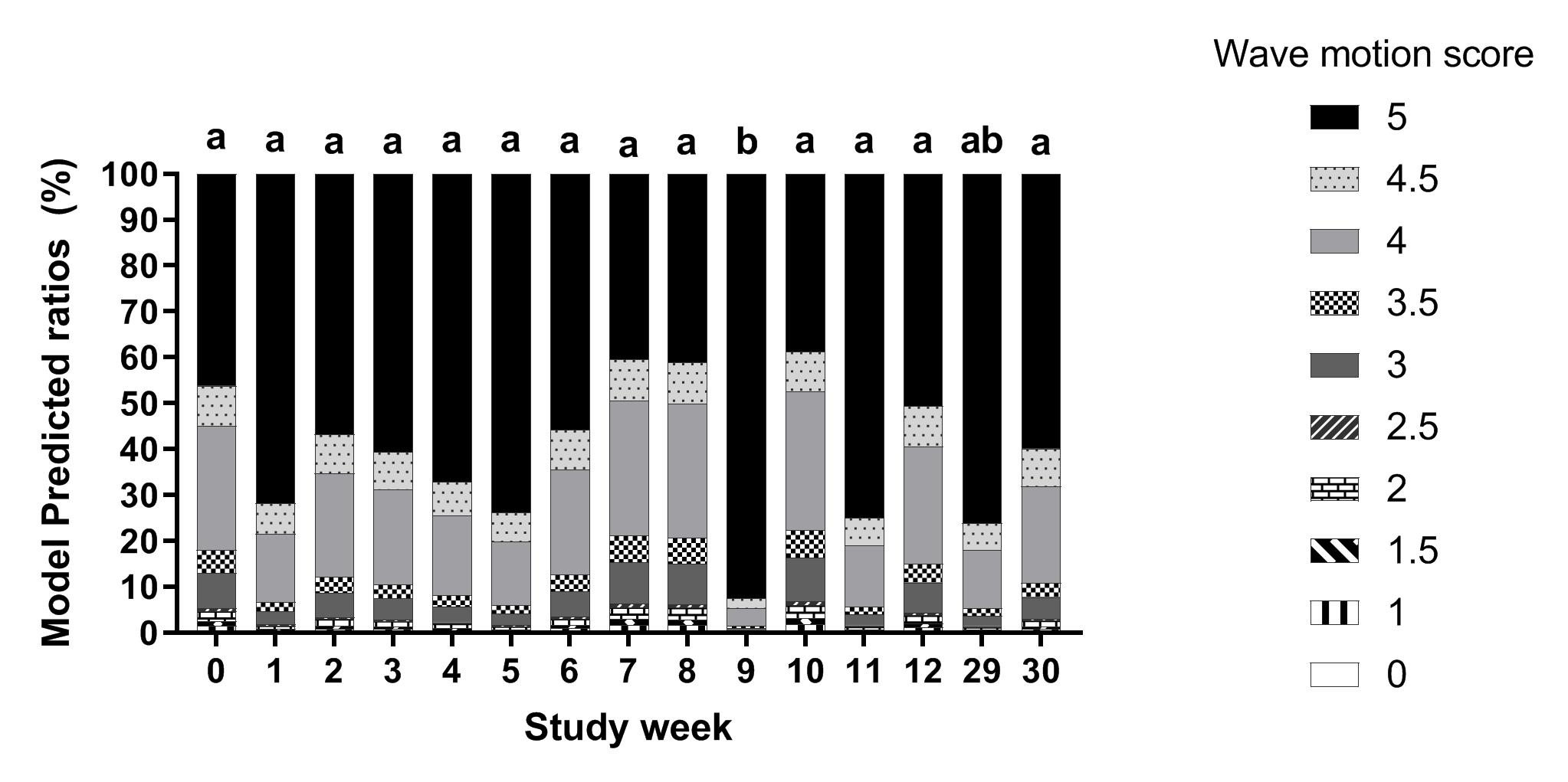

Supplement: Supplementary file 4 — Supplementary Figure S4 [file 41598_2020_66594_MOESM4_ESM.jpg]

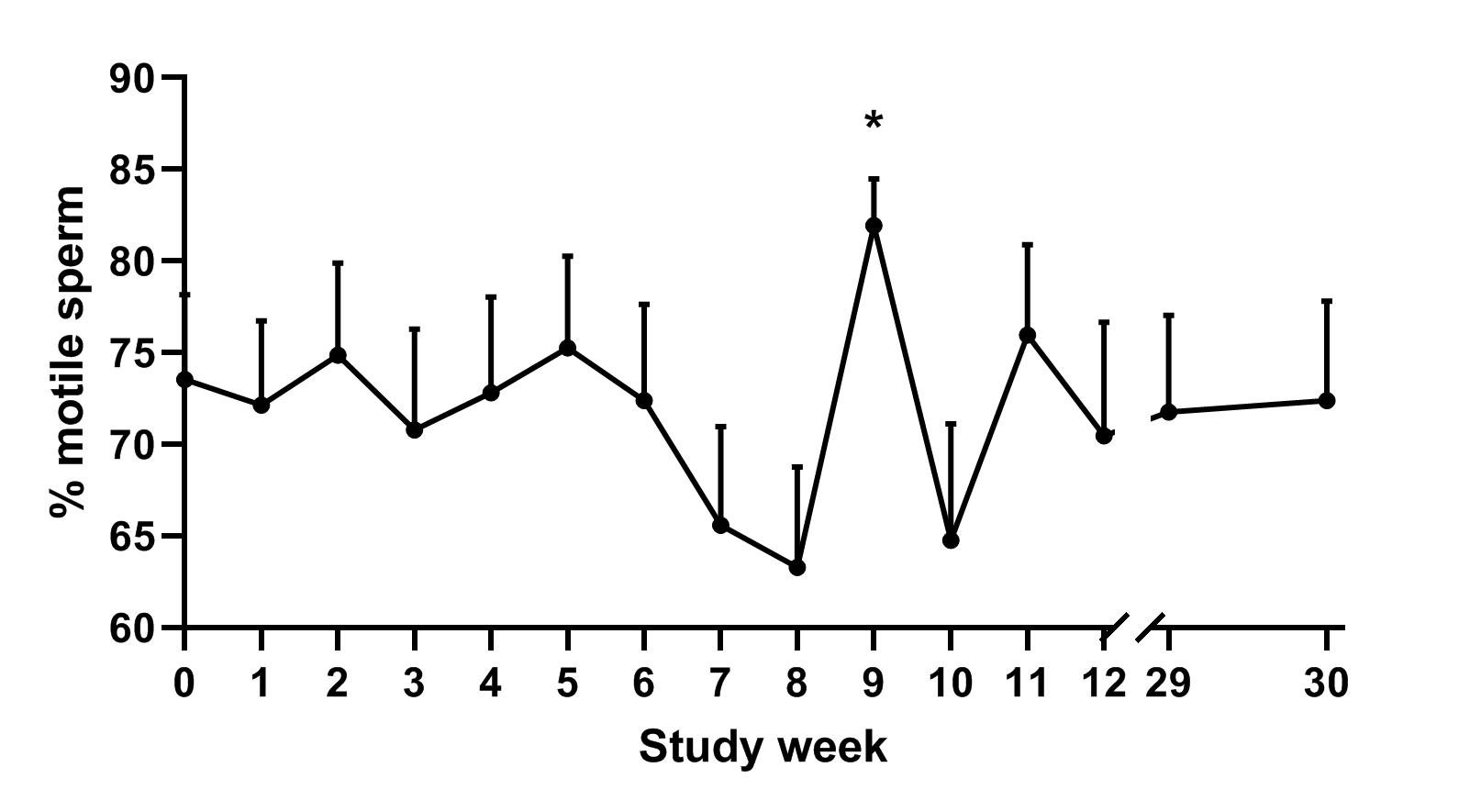

Supplement: Supplementary file 5 — Supplementary Figure S5 [file 41598_2020_66594_MOESM5_ESM.jpg]

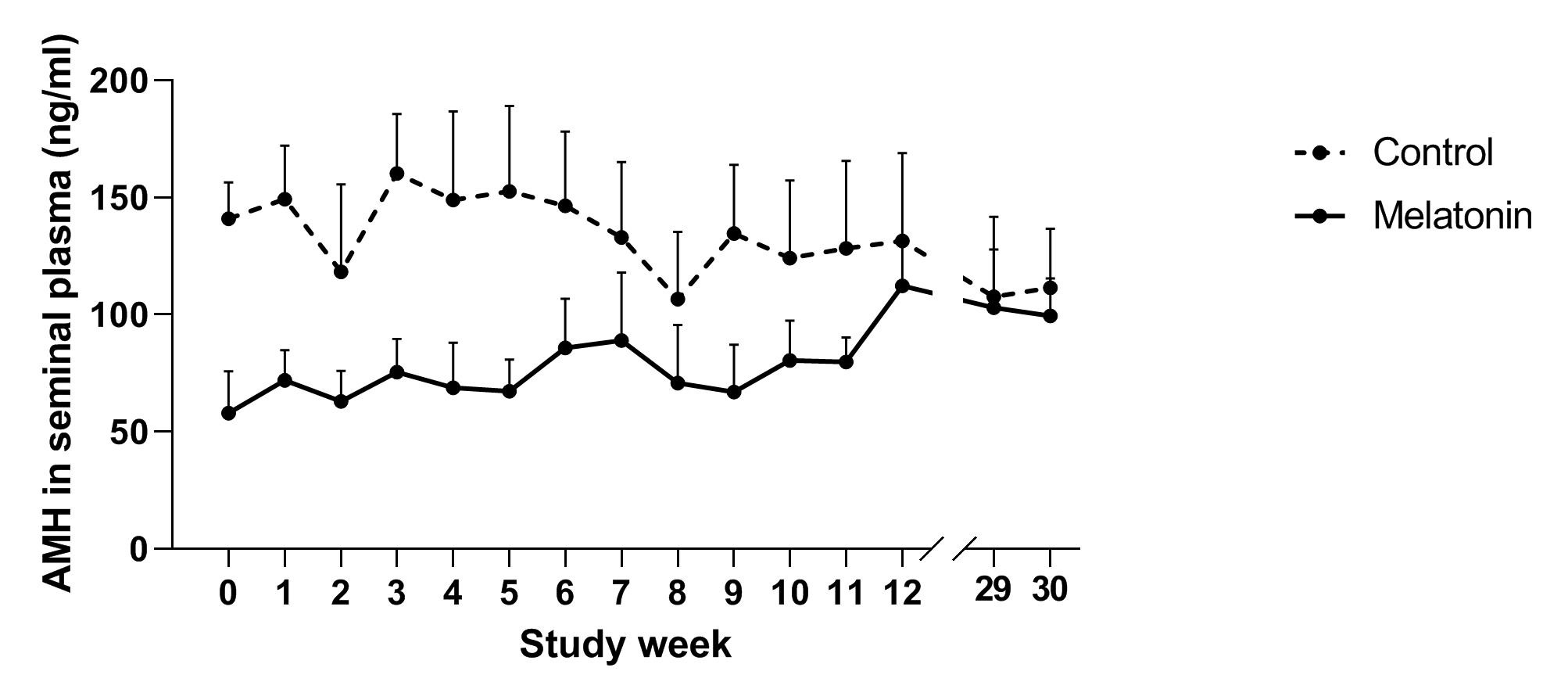

Supplement: Supplementary file 6 — Supplementary Figure S6 [file 41598_2020_66594_MOESM6_ESM.jpg]

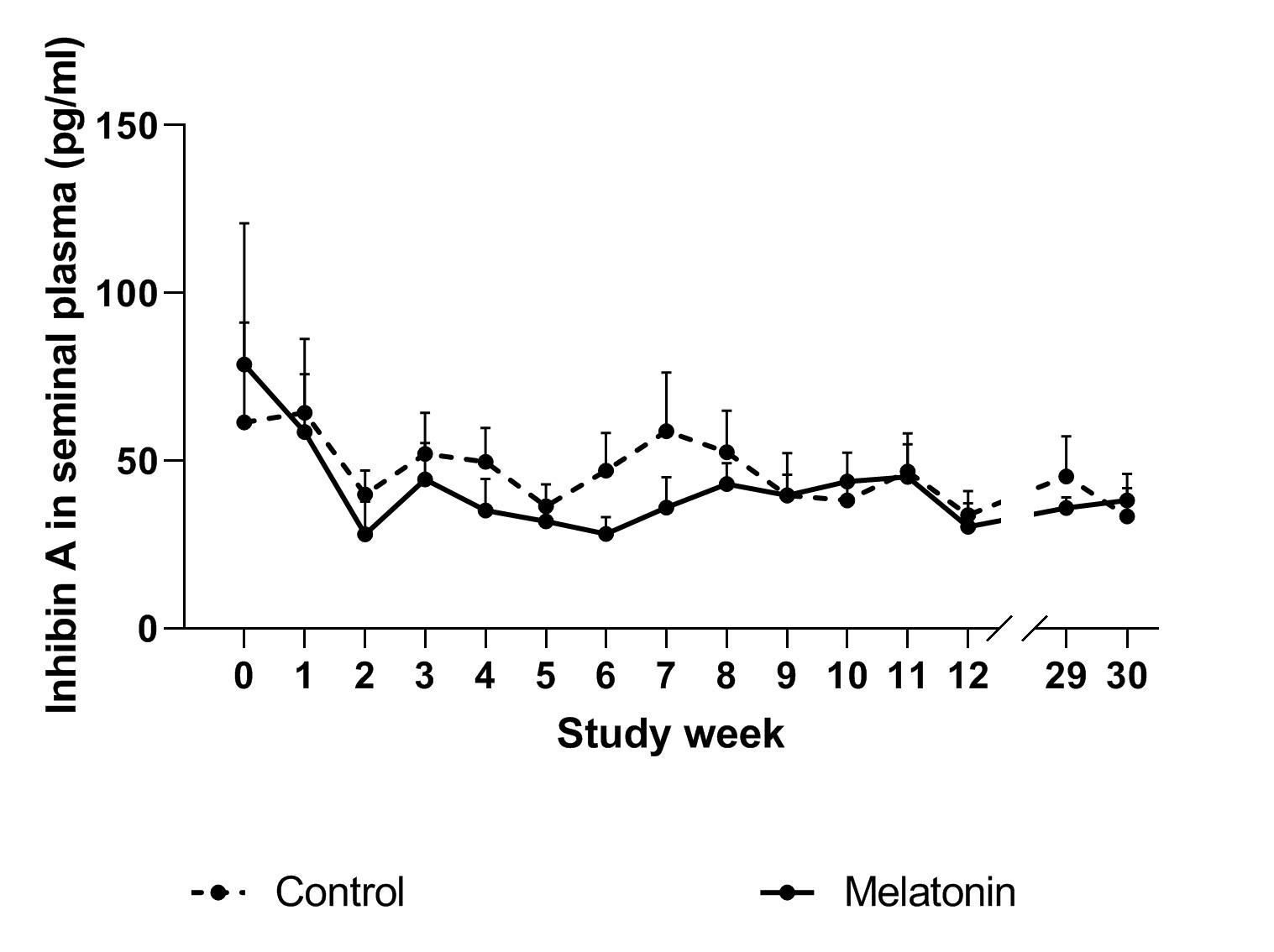

Supplement: Supplementary file 7 — Supplementary Figure S7 [file 41598_2020_66594_MOESM7_ESM.jpg]
