# Supplementary material for: Exogenous melatonin advances the ram breeding season and increases testicular function
Source: Sci Rep. 2020 Jun 16;10:9711. doi: 10.1038/s41598-020-66594-6 (PMC7297710; doi:10.1038/s41598-020-66594-6)
Supplement: Supplementary file 8 — Supplementary information [file 41598_2020_66594_MOESM8_ESM.docx]

**Supplementary Information**

Exogenous melatonin advances the ram breeding season and increases testicular function

**Author list and Affiliations**

K.R. Pool^1^, J.P. Rickard^1^, T. Pini^1^ and S.P. de Graaf^1^

*^1^*The University of Sydney, Faculty of Science, School of Life and Environmental Sciences, Sydney NSW 2006, Australia

Corresponding author email: [kelsey.pool@sydney.edu.au](mailto:kelsey.pool@sydney.edu.au)

**Supplementary Figure S1**. The percentage of successful ejaculations from melatonin and control Poll Dorset rams (n=14) from study week 0 (1week post-implantation) to study week 30 (following breeding season). Weekly values are presented as means ± S.E.M. * Indicates significant difference from week 0 within treatment group (P<0.05).

**Supplementary Figure S2**. The model predicted ratios of obtaining a body condition score (BCS) from 2.5-4.5 from melatonin-treated and control rams (n= 31). Values are presented as the proportion of rams in each treatment group assigned to each BCS category. Different letters indicate significant differences (P<0.05).

**Supplementary Figure S3**. The model predicted ratios of obtaining an ejaculate score from 0-5 from melatonin-treated and control rams (n= 31). Values are presented as the proportion of ejaculates from rams in each treatment group assigned to each consistency score category. Different letters indicate significant differences (P<0.05).

**Supplementary Figure S4**. The model predicted ratios of obtaining a wave motion score from 0-5 from Week 0 (1week post-implantation) to study week 30 (following breeding season) in all rams (n= 31).. Weekly values are presented as the percentage of ejaculates assigned each wave motion score. Different letters indicate significant difference between study weeks (P<0.05).

**Supplementary Figure S5**. The percentage of motile spermatozoa from study week 1 (1week post-implantation) to study week 30 (following breeding season) in all rams (n= 31). Weekly values are presented as means ± S.E.M. * Indicates significant difference from week 0 (P<0.05).

**Supplementary Figure S6**. The concentration of Anti-Mullerian Hormone (AMH) in the seminal plasma of melatonin-treated and control rams from study week 1 (1week post-implantation) to study week 30 (following breeding season). Data is based off the 18 rams that collected from week 0 (Melatonin n =9, Control n =9). Weekly values are presented as means ± S.E.M. * Indicates significant difference from week 0 (P<0.05).

**Supplementary Figure S7**. The concentration of Inhibin A in the seminal plasma of melatonin-treated and control rams from study week 1 (1week post-implantation) to study week 30 (following breeding season). Data is based off the 18 rams that collected from week 0 (Melatonin n =9, Control n =9).Weekly values are presented as means ± S.E.M. * Indicates significant difference from week 0 (P<0.05).
